# Supplementary material for: Association of Glycemic Index, Glycemic Load, and Carbohydrate Intake with Antral Follicle Counts Among Subfertile Females
Source: Nutrients. 2025 Jan 21;17(3):382. doi: 10.3390/nu17030382 (PMC11820038; doi:10.3390/nu17030382)
Supplement: Supplementary file 1 [file nutrients-17-00382-s001.zip › nutrients-3377791-supplementary.pdf]

nutrients

MDPI

Supplementary Table S1: Comparison of demographic and reproductive characteristics of study included and excluded participants[a,b,c]

		Total	Included	Excluded	P-value[c]
n		877	653	224	
Demographic characteristics					
Age (y)		35.0 (32.0-38.0)	35.0 (32.0-38.0)	35.0 (32.0-39.0)	0.16
BMI (kg/m^2)		23.5 (21.3-26.7)	23.4 (21.2-26.4)	23.8 (21.6-28.4)	0.04
Race and ethnicity,					0.007
non-Hispanic white, n (%)		677 (77.2)	514 (78.7)	163 (72.8)	
non-Hispanic Black, n (%)		36 (4.1)	27 (4.1)	9 (4.0)	
non-Hispanic Asian, n (%)		85 (9.7)	66 (10.1)	19 (8.5)	
non-Hispanic Other, n (%)		25 (2.9)	13 (2.0)	12 (5.4)	
Hispanic, any race, n (%)		34 (3.9)	32 (4.9)	2 (5.9)	
Smoking Status, never, n (%)		636 (72.3)	485 (74.5)	151 (73.7)	0.81
Education, higher than college graduation, n (%)		678 (91.4)	568 (91.9)	94 (88.7)	0.48
Reproductive History					
Previous infertility examination, n (%)		697 (84.0)	524 (83.0)	173 (86.9)	0.19
Previous infertility treatment, n (%)		422 (56.1)	311 (54.6)	111 (61.0)	0.12
History of past pregnancy, n (%)		380 (44.5)	278 (42.8)	102 (50.0)	0.07
Primary infertility diagnosis, n (%)					0.0005
Male factor		208 (24.4)	159 (24.5)	49 (24.0)	
Female factor	DOR	95 (11.1)	59 (9.1)	36 (17.7)	
	Endometriosis	40 (4.7)	24 (3.7)	16 (7.8)	
	Ovulatory	95 (11.1)	76 (11.7)	30 (13.8)	
	Tubal	46 (5.4)	33 (5.1)	19 (9.3)	
	Uterine	12 (1.4)	11 (1.7)	1 (0.5)	
Unexplained		357 (41.8)	288 (44.3)	69 (33.8)	
Antral follicle count		12.0 (8.0, 18.0)	13.0 (9.0, 18.0)	11.0 (7.0, 17.0)	0.006
Follicle stimulating hormone (mIU/mL)		6.9 (5.9, 8.4)	6.8 (5.9, 8.3)	7.0 (6.1, 8.7)	0.13
Anti-Müllerian hormone (ng/mL)		2.2 (1.0, 4.4)	2.1 (1.0, 4.3)	3.0 (1.3, 5.3)	0.31

[a]BMI: body mass index, DOR: Diminished ovarian reserve,

[b]Data is presented as median (interquartile range) for continuous variables or n (%) for categorical variables.

[c]P-value was calculated by using the Kusmal-Willis nonlinearity test for continuous variables, and chi-square test for categorical variables.

Supplementary Table S2: Demographic and reproductive characteristics of study participants, overall and according to energy density carbohydrate[a,b]

	Total	Tertile1 (Lowest)	Tertile2 (Middle)	Tertile3 (Highest)
n	653	217	218	218
Carbohydrate energy density, median (Range)	48.27 (16.58-69.98)	40.47 (16.58-44.68)	48.26 (44.70-51.67)	55.64 (51.69-69.98)
Demographic characteristics				
Age (y)	35.0 (32.0-38.0)	36.0 (32.0-39.0)	34.0 (31.0-38.0)	35.0 (32.0-38.0)
BMI (kg/m^2)	23.4 (21.2-26.4)	23.9 (21.7-27.0)	23.4 (21.4-26.5)	22.7 (20.9-25.7)
Race and ethnicity,				
Non-Hispanic white, n (%)	514 (78.7)	174 (80.2)	181 (83.0)	159 (72.9)
Non-Hispanic Black, n (%)	27 (4.1)	6 (2.8)	9 (4.1)	12 (5.5)
Non-Hispanic Asian, n (%)	66 (10.1)	12 (5.5)	16 (7.3)	38 (17.4)
Non-Hispanic Other, n (%)	13 (2.0)	7 (3.2)	3 (1.4)	3 (1.4)
Hispanic, any race, n (%)	32 (4.9)	18 (8.3)	8 (3.7)	6 (2.8)
Smoking status, never, n (%)	485 (74.3)	151 (69.6)	154 (70.6)	180 (82.6)
Education, higher than college graduation, n (%)	568 (87.0)	184 (84.8)	194 (89.0)	190 (87.2)
Physical activity (hr/week)	5.0 (2.5-9.5)	5.0 (2.0-10.0)	5.1 (2.5-10.0)	5.1 (2.5-9.0)
Multivitamin intake, n (%)	554 (84.8)	177 (81.6)	188 (86.2)	189 (86.7)
Total calorie intake (kcal/day)	1682.3 (1363.1-2058.5)	1694.4 (1364.8-2043.1)	1645.5 (1324.4-2076.5)	1698.4 (1363.7-2027.1)
Protein (Energy density [%])	16.5 (14.9-18.6)	17.9 (15.9-20.0)	16.8 (15.3-18.6)	15.5 (14.0-16.8)
Total fat (Energy density [%])	33.0 (29.5-37.4)	38.7 (35.2-42.9)	33.4 (31.0-35.7)	28.6 (25.9-30.9)
Fiber (g/day)	19.9 (15.3-26.4)	17.4 (13.3-23.3)	19.7 (15.5-26.6)	22.0 (17.0-30.1)
Total sugar (g/day)	82.4 (62.2, 109.5)	67.1 (51.2-84.6)	83.5 (65.8-104.3)	105.8 (75.2-132.3)
Alcohol (mg/day)	4.7 (1.4-12.5)	8.7 (3.2-15.4)	5.5 (2.1-12.7)	2.7 (0.6-6.7)
Caffeine (mg/day)	105.0 (44.7-171.7)	110.7 (58.4-243.4)	109.1 (42.5-175.0)	95.4 (28.7-138.3)
Glycemic Index	50.5 (48.0-52.9)	48.5 (46.2-52.1)	50.5 (48.7-52.8)	51.3 (49.6-53.9)
Glycemic load	109.9 (75.8-129.9)	79.3 (61.8-102.1)	101.4 (80.3-127.5)	124.2 (99.4-154.3)
Reproductive History				
Previous infertility examination, n (%)	524 (80.3)	173 (79.7)	166 (76.2)	185 (84.9)
Previous infertility treatment, n (%)	311 (47.6)	90 (41.5)	102 (46.8)	119 (54.6)
History of past pregnancy, n (%)	278 (42.6)	99 (45.6)	89 (40.8)	90 (41.3)
Primary infertility diagnosis, n (%)				

Male factor		159 (24.5)	53 (24.7)	43 (19.8)	63 (28.9)
Female factor	DOR	59 (9.1)	20 (9.3)	15 (6.9)	24 (11.0)
	Endometriosis	24 (3.7)	7 (3.3)	8 (3.7)	9 (4.1)
	Ovulatory	76 (11.7)	26 (12.1)	30 (13.8)	20 (9.2)
	Tubal	33 (5.1)	8 (3.7)	14 (6.5)	11 (5.1)
	Uterine	11 (1.7)	8 (3.7)	2 (0.9)	1 (0.5)
Unexplained		288 (44.3)	93 (43.3)	105 (48.4)	90 (41.3)

[a]BMI: body mass index, DOR: Diminished ovarian reserve,

[b]Data is presented as median (interquartile range) for continuous variables or n (%) for categorical variables.

Supplementary Table S3: Demographic and reproductive characteristics of study participants, overall and according to energy density glycemic load[a,b]

	**Total**	**Tertile1 (Lowest)**	**Tertile2 (Middle)**	**Tertile3 (Highest)**
n	653	217	218	218
Glycemic load, median (Range)	100.88 (19.97-306.67)	66.62 (19.97-83.68)	100.83 (83.72-118.06)	143.98 (118.55-306.67)
Demographic characteristics				
Age (y)	35.0 (32.0-38.0)	35.0 (32.0-38.0)	35.0 (32.0-38.0)	35.0 (32.0-38.0)
BMI (kg/m^2)	23.4 (21.2-26.4)	23.7 (21.5-26.4)	23.3 (21.3-26.2)	23.1 (20.9-26.4)
Race and ethnicity,				
Non-Hispanic white, n (%)	514 (78.7)	160 (73.7)	183 (83.9)	171 (78.4)
Non-Hispanic Black, n (%)	27 (4.1)	9 (4.2)	7 (3.2)	11 (5.1)
Non-Hispanic Asian, n (%)	66 (10.1)	23 (10.6)	17 (7.8)	26 (11.9)
Non-Hispanic Other, n (%)	13 (2.0)	6 (2.8)	5 (2.3)	2 (0.9)
Hispanic, any race, n (%)	32 (4.9)	18 (8.3)	6 (2.8)	8 (3.7)
Smoking status, never, n (%)	485 (74.3)	155 (71.4)	169 (77.5)	161 (73.9)
Education, higher than college graduation, n (%)	568 (87.0)	180 (83.0)	198 (90.8)	190 (87.2)
Physical activity (hr/week)	5.0 (2.5-9.5)	5.0 (1.5-9.5)	5.1 (2.5-8.7)	5.5 (3.0-12.0)
Multivitamin intake, yes, n (%)	554 (84.8)	184 (84.8)	184 (84.4)	186 (85.3)
Total calorie intake (kcal/day)	1682.3 (1363.1-2058.5)	1231.6 (1037.9-1499.2)	1632.8 (1463.2-1828.2)	2215.4 (1945.4-2600.2)
Carbohydrates (Energy density [%])	48.3 (43.0-53.5)	43.5 (37.8-48.8)	48.5 (43.9-52.7)	52.4 (48.0-56.3)
Protein (Energy density [%])	16.5 (14.9-18.6)	18.0 (15.6-19.9)	16.5 (14.8-18.3)	15.7 (14.4-17.2)
Total fat (Energy density [%])	33.0 (29.5-37.4)	35.4 (31.1-40.2)	33.0 (29.6-37.4)	31.6 (28.1-34.5)
Fiber (g/day)	19.9 (15.3-26.4)	14.8 (11.3-18.3)	19.5 (16.0-24.8)	27.3 (22.5-34.4)
Total sugar (g/day)	82.4 (62.2-109.5)	56.5 (47.6-67.6)	83.8 (69.8-98.2)	120.6 (97.9-149.6)
Alcohol (mg/day)	4.7 (1.4-12.5)	4.7 (1.4-12.7)	4.9 (1.6-12.1)	4.8 (1.2-13.2)

Caffeine (mg/day)	105.0 (44.7-171.7)	101.4 (44.0-154.7)	108.1 (48.0-170.3)	109.4 (38.9-217.5)
Glycemic Index	50.5 (48.0-52.9)	48.8 (46.1-51.2)	50.6 (48.3-52.9)	51.6 (49.6-54.2)
Reproductive History				
Previous infertility examination, yes, n (%)	524 (80.3)	176 (81.1)	175 (80.3)	173 (79.4)
Previous infertility treatment, yes, n (%)	311 (47.6)	102 (47.0)	104 (47.7)	105 (48.2)
History of past pregnancy yes, n (%)	278 (42.6)	96 (44.2)	89 (40.8)	93 (42.7)
Primary infertility diagnosis				
Male factor	159 (24.5)	52 (24.3)	53 (24.3)	54 (24.8)
Female factor DOR	59 (9.1)	19 (8.9)	19 (8.7)	21 (9.6)
Endometriosis	24 (3.7)	8 (3.7)	5 (2.3)	11 (5.1)
Ovulatory	76 (11.7)	24 (11.2)	25 (11.5)	27 (12.4)
Tubal	33 (5.1)	6 (2.8)	11 (5.1)	16 (7.3)
Uterine	11 (1.7)	4 (1.8)	4 (1.8)	3 (1.4)
Unexplained	288 (44.3)	101 (47.2)	101 (46.3)	86 (39.5)

[a]BMI: body mass index, DOR: Diminished ovarian reserve,

[b]Data is presented as median (interquartile range) for continuous variables or n (%) for categorical variables.

Supplementary Table S4: Spearman correlation of glycemic index, load, and carbohydrate intake among 653 participants

	Glycemic index	Glycemic load	Carbohydrate
Glycemic index	1		
Glycemic load	0.37	1	
Carbohydrate	0.32	0.49	1

Supplementary Table S5: Food Sources of Carbohydrates among study participants

	Total
**High-quality carbohydrate**	34.13
Whole grains	16.85
Whole fruits	9.31
Nonstarchy vegetables	5.77
Legumes	2.20
**Low-quality carbohydrate**	44.88
Refined grains	19.72
Added sugar	15.67
Potato	4.90

Fruits juice	3.22
Other starchy vegetables	1.37
**Others food groups**	20.55
Dairy	12.65
Others	7.59
Nuts	0.31

[a] Food source for carbohydrate (%) in this study participants

Supplementary Table S6: Comparison of food sources for carbohydrates with consideration to carbohydrate quality between EARTH participants and NHANES data in the corresponding period[a]

%	EARTH	NHANES
High-quality carbohydrate	21.27	8.62
Whole grains	10.67	2.67
Whole fruits	4.45	3.25
Nonstarchy vegetables	4.38	1.78
Legumes	1.77	0.93
Low-quality carbohydrate	32.57	36.75
Refined grains	13.54	15.70
Fruits juice	1.63	3.19
Potato	3.88	2.49
Other starchy vegetables	0.71	0.60
Added sugar	12.81	14.78

[a]The table shows the estimated percentage of energy from carbohydrates. NHANES data came from adults aged 20 years or older [29]. The value was calculated the average of 5 surveys from 2007 to 2016. The survey-weighted means of energy intake were displayed.

Supplementary Table S7: Comparison of food sources for carbohydrate intakes among EARTH participants based on history of infertility examination[a]

Serving/day	Total	History of infertility examination		Difference in median	P-value
		Yes	No		
n	653	524	107		
High-quality carbohydrate	6.21	6.26	5.91	0.35	0.13
Whole grains	1.17	1.15	1.18	-0.03	0.78
Whole fruits	1.09	1.12	0.89	0.23	0.04
Nonstarchy vegetables	3.19	3.24	3.06	0.18	0.15
Legumes	0.26	0.26	0.24	0.02	0.69
Low-quality carbohydrate	4.33	4.30	4.44	-0.14	0.90
Refined grains	1.42	1.40	1.55	-0.15	0.37

Fruits juice	0.16	0.16	0.16	0.00	0.95
Potato	0.24	0.18	0.24	-0.06	0.05
Other starchy vegetables	0.16	0.16	0.12	0.04	0.22
Added sugar	1.81	1.77	1.93	-0.16	0.79

[a] Each food source is displayed as median value of serving/day. P-values were obtained by using Kruskal-Wallis test.

Supplementary Table S8: Multivariable-adjusted association of glycemic index, glycemic load, and carbohydrate intake with adjusted antral follicle count (AFC>30 as AFC=30) among 579 women who had numeric AFC measurement[a,b,c]

		n	AFC, Relative Difference in Mean (95% CI) (%)	
	Index Range		Age + calorie adjusted model	MV model
Glycemic index	30.92-48.96	195	Reference	Reference
	48.97-51.98	194	2.1 (-3.3, 7.8)	1.7 (-3.8, 7.5)
	51.99-60.20	190	4.5 (-0.9, 10.3)	4.8 (-0.9, 10.8)
	P, trend		p=0.11	p=0.10
Glycemic load	19.97-83.68	188	Reference	Reference
	83.72-118.06	200	-1.3 (-6.8, 4.6)	-1.1 (-6.8, 5.0)
	118.55-306.67	191	0.7 (-6.9, 8.8)	0.5 (-7.4, 9.1)
	P, trend		p=0.87	p=0.90
Carbohydrate (Energy density) (%)	16.58-44.68	191	Reference	Reference
	44.70-51.67	194	-3.6 (-8.7, 1.7)	-4.5 (-9.6, 1.0)
	51.69-69.98	194	-3.6 (-8.7, 1.7)	-5.6 (-10.9, 0.0)
	P, trend		p=0.18	p=0.05

[a]AFC: antral follicle count, MV: multivariable

[b]The Poisson regression models were adjusted for age, BMI, physical activity, calorie intake, alcohol intake, caffeine intake, race and ethnicity (non-Hispanic white[reference], non-Hispanic black, non-Hispanic Asian, non-Hispanic other, Hispanic), smoking status (Never smoker[reference], ever smoker, missing), education status (higher than college graduation), and multivitamin supplement use (yes, no[reference], missing).

[c] In the models, any AFC value greater than 30 was capped at 30.

nutrients

MDPI

Supplementary Table S9: Multivariable-adjusted association of glycemic index, glycemic load, carbohydrate, and fiber intake (tertile) with adjusted antral follicle count (AFC>30 as AFC=30) among 579 women who had numeric AFC measurement according to previous infertility examination status[a,b]

	Women with history of infertility examination				Women without history of infertility examination				
Index Range	n	AFC Median (IQR)	Age + calorie adjusted model	MV model+AFC adjustment (30)[c]	n	AFC Median (IQR)	Age + calorie adjusted model	MV model+AFC adjustment (30)[c]	$P_{hetero-geneity}$ [d]
Glycemic index									
30.92-48.96	165	12.0 (8.0, 17.0)	Reference	Reference	24	11.0 (8.0, 17.5)	Reference	Reference	0.11
48.97-51.98	154	13.0 (10.0, 18.0)	3.0 (-2.9, 9.3)	2.5 (-3.6, 9.0)	32	12.5 (9.0, 18.0)	-3.9 (-17.6, 12.0)	-1.8 (-17.2, 16.4)	
51.99-60.20	146	13.0 (9.0, 19.0)	3.1 (-2.8, 9.5)	2.7 (-3.5, 9.2)	37	13.0 (11.0, 20.0)	11.0 (-3.9, 28.2)	19.0 (1.2, 39.9)	
P, trend			p=0.30	p=0.39			p=0.10	p=0.01	
Glycemic load									
19.97-83.68	154	13.0 (9.0, 18.0)	Reference	Reference	28	14.5 (10.0, 19.0)	Reference	Reference	0.02
83.72-118.06	160	12.0 (9.0, 18.0)	0.6 (-5.6, 7.3)	1.0 (-5.4, 7.9)	36	12.5 (10.0, 18.5)	-10.0 (-22.2, 4.1)	-6.2 (-19.9, 9.9)	
118.55-306.67	151	13.0 (9.0, 18.0)	2.3 (-6.1, 11.5)	2.2 (-6.6, 11.9)	29	11.0 (9.0, 17.0)	-14.9 (-30.8, 4.6)	-8.3 (-27.1, 15.3)	
P, trend			p=0.60	p=0.63			p=0.13	p=0.46	
Carbohydrate (Energy density) (%)									
16.58-44.68	153	13.0 (10.0, 18.0)	Reference	Reference	33	11.0 (8.0, 17.0)	Reference	Reference	0.20
44.70-51.67	145	13.0 (9.0, 18.0)	-5.2 (-10.8, 0.7)	-6.1 (-11.7, 0.0)	36	13.0 (9.0, 18.5)	0.2 (-12.1, 14.2)	-2.5 (-15.3, 12.3)	
51.69-69.98	167	12.0 (9.0, 18.0)	-5.9 (-11.2, -0.2)	-8.3 (-14.0, -2.3)	24	15.5 (10.0, 19.0)	5.3 (-8.7, 21.5)	9.9 (-6,8, 29.5)	
P, trend			p=0.05	p=0.008			p=0.50	p=0.32	

aAFC: antral follicle count, IQR: interquartile range, MV: multivariable

bThe Poisson regression models were adjusted for age, BMI, physical activity, calorie intake, alcohol intake, caffeine intake, race and ethnicity (non-Hispanic white[reference], non-Hispanic black, non-Hispanic Asian, non-Hispanic other, Hispanic), smoking status (Never smoker[reference], ever smoker, missing), education status (higher than college graduation), and multivitamin supplement use (yes, no[reference], missing).

cIn the models, any AFC value greater than 30 was capped at 30.

dHeterogeneity by infertility status was evaluated by adding the interaction term for infertility status and exposures in the models.

Supplementary Table S10: Age, calorie-adjusted odds ratios of glycemic index, glycemic load, and carbohydrate with polycystic ovary morphology according to antral follicle count[a,b]

	Index Range	n (%)	Women with history of infertility examination	n (%)	Women without history of infertility examination	$P_{heterogeneity}$[c]
Glycemic index	30.92-48.96	17/182 (9.3)	Reference	2/28 (7.1)	Reference	0.57
	48.97-51.98	12/173 (6.9)	0.63 (0.28, 1.38)	4/36 (11.1)	1.08 (0.17, 6.85)	
	51.99-60.20	23/167 (13.8)	1.30 (0.65, 2.59)	6/43 (14.0)	2.27 (0.38, 13.70)	
	P, trend		p=0.45		p=0.32	
Glycemic load	19.97-83.68	19/175 (10.9)	Reference	6/34 (17.7)	Reference	0.53
	83.72-118.06	15/174 (8.6)	0.58 (0.27, 1.26)	3/39 (7.7)	0.93 (0.15, 5.90)	
	118.55-306.67	18/173 (10.4)	0.40 (0.14, 1.14)	3/34 (8.8)	3.18 (0.25, 41.01)	
	P, trend		p=0.08		p=0.38	
Carbohydrate (Energy density) (%)	16.58-44.68	19/173 (11.0)	Reference	5/38 (13.2)	Reference	0.63
	44.70-51.67	23/166 (13.9)	1.09 (0.55, 2.14)	4/39 (10.3)	0.68 (0.16, 2.86)	
	51.69-69.98	10/183 (5.5)	0.36 (0.15, 0.82)	3/30 (10.0)	0.78 (0.16, 3.79)	
	P, trend		p=0.02		p=0.72	

[a]The logistic regression models were adjusted for age and calorie intake only, due to the small number of cases.

[b]Polycystic ovarian morphology was defined as follicle number per ovary ≥ 20 in at least one ovary.

[c]Heterogeneity by infertility status was evaluated by adding the interaction term for infertility status and exposures in the models.

nutrients

MDPI

Supplementary Table S11: Multivariable-adjusted odds ratios of glycemic index, glycemic load, and carbohydrate with diminished ovarian reserve[a,b,c]

	Index Range	n/women (%)	Odds ratio (95% CI) (%) Age + calorie	MV model
Glycemic index	30.92-48.96	26/217 (12.0)	Reference	Reference
	48.97-51.98	19/219 (8.7)	0.84 (0.44, 1.60)	0.82 (0.42, 1.61)
	51.99-60.20	23/217 (10.6)	1.16 (0.62, 2.16)	1.05 (0.54, 2.03)
	P, trend		p=0.70	p=0.93
Glycemic load	19.97-83.68	28/217 (12.9)	Reference	Reference
	83.72-118.06	19/218 (8.7)	0.69 (0.35, 1.36)	0.66 (0.32, 1.36)
	118.55-306.67	21/218 (9.6)	0.84 (0.35, 2.04)	0.89 (0.35, 2.28)
	P, trend		p=0.67	p=0.79
Carbohydrate	16.58-44.68	24/217 (11.1)	Reference	Reference
(Energy density)	44.70-51.67	20/218 (9.2)	1.02 (0.53, 1.96)	1.08 (0.55, 2.15)
(%)	51.69-69.98	24/218 (11.0)	1.20 (0.64, 2.25)	1.42 (0.71, 2.85)
	P, trend		p=0.57	p=0.32

[a]MV: multivariable

[b]The logistic regression models were adjusted for age, BMI, physical activity, calorie intake, alcohol intake, caffeine intake, race and ethnicity (non-Hispanic white[ref], non-Hispanic black, non-Hispanic Asian, non-Hispanic other, Hispanic), smoking status (Never smoker[ref], ever smoker, missing), education status (higher than college graduation), and multivitamin supplement use (yes, no[ref], missing).

[c] Diminished ovarian reserve was defined as antral follicle count less than 7.

nutrients

MDPI

Supplementary Table S12: Age, calorie-adjusted odds ratios of glycemic index, glycemic load, and carbohydrate intake with diminished ovarian reserve[a,b,c]

	Index Range	n (%)	Women with history of infertility examination	n	Women without history of infertility examination	$P_{heterogeneity}$[c]
Glycemic index	30.92-48.96	18/183 (9.8)	Reference	3/28 (10.7)	Reference	0.65
	48.97-51.98	15/174 (8.6)	0.88 (0.43, 1.77)	3/36 (8.3)	1.61 (0.22, 11.65)	
	51.99-60.20	19/167 (11.4)	1.09 (0.55, 2.18)	3/43 (7.0)	1.29 (0.20, 8.45)	
	P, trend		p=0.84		p=0.79	
Glycemic load	19.97-83.68	18/176 (10.2)	Reference	2/34 (5.9)	Reference	0.92
	83.72-118.06	15/175 (8.6)	0.64 (0.31, 1.34)	2/39 (5.1)	0.81 (0.08, 7.95)	
	118.55-306.67	19/173 (11.0)	0.67 (0.25, 1.79)	5/34 (14.7)	3.07 (0.25, 38.31)	
	P, trend		p=0.39		p=0.30	
Carbohydrate (Energy density) (%)	16.58-44.68	18/173 (10.4)	Reference	2/38 (5.3)	Reference	0.75
	44.70-51.67	20/166 (12.1)	0.97 (0.47, 1.98)	3/39 (7.7)	3.42 (0.38, 31.08)	
	51.69-69.98	14/185 (7.6)	1.05 (0.53, 2.08)	4/30 (13.3)	5.09 (0.64, 40.67)	
	P, trend		p=0.89		p=0.12	

[a]The logistic regression models were adjusted for age and calorie intake only, due to the small number of cases.
[b]Diminished ovarian reserve was defined as antral follicle count less than 7.
[c]Heterogeneity by infertility status was evaluated by adding the interaction term for infertility status and exposures in the models.

Supplementary Figure S1

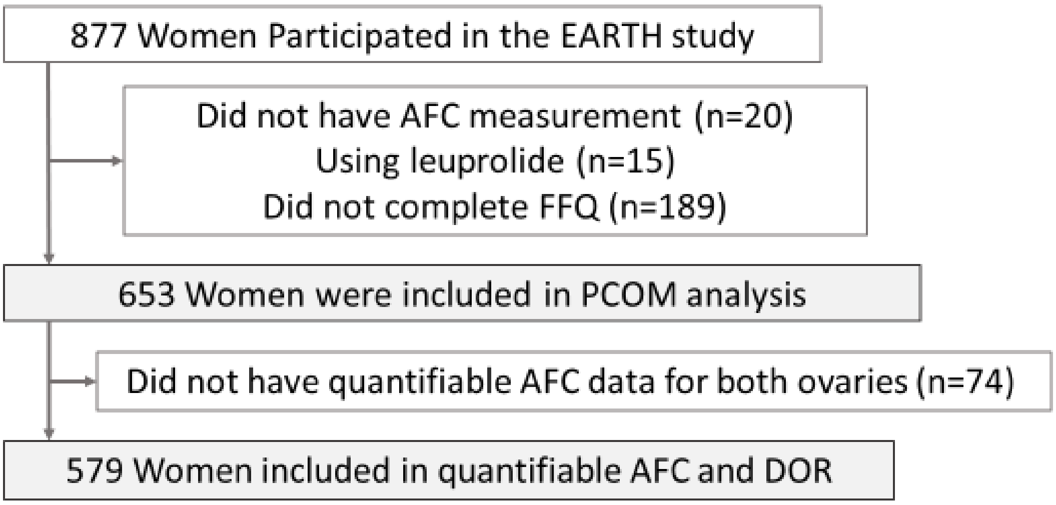

Supplementary Figure S1: Flowchart of participant selection for the analysis.
